# Historical overview

**DOI:** 10.1186/ar4220

**Published:** 2013-07-11

**Authors:** Leonard H Calabrese

**Affiliations:** 1 Professor of Medicine, Cleveland Clinic Lerner College of Medicine, RJ Fasenmyer Chair of Clinical Immunology, Cleveland Clinic Foundation, Cleveland, Ohio, USA

## 

Medical understanding systemic lupus erythematosus (SLE) has evolved dramatically since its first reference during the 1100s, through the neoclassic period of the 1800s when researchers began to refine the definition, and into the modern era with transformative laboratory investigations and discoveries altering the diagnosis and treatment. Weaved into this historic overview are descriptions of the most important contributions of the leading practitioners and researchers.

Additional file 1 contains an edited transcript of the full presentation.

## Competing interests

LHC has received consulting fees from Amgen, BMS, Centecor, Genentech/Roche, Pfizer, Sanofi Aventis and Savient.

## Supplementary Material

Additional file 1 Click here for file

