# Supplementary material for: Historical overview
Source: Arthritis Res Ther. 2013 Jul 11;15(Suppl 2):A2. doi: 10.1186/ar4220 (PMC3891298; doi:10.1186/ar4220)
Supplement: Additional file 1 [file ar4220-S1.pdf]

Presentation

Historical overview

Leonard H Calabrese

Professor of Medicine, Cleveland Clinic Lerner College of Medicine, RJ Fasenmyer Chair of Clinical Immunology, Cleveland Clinic Foundation, Cleveland, Ohio, USA

Abstract

Medical understanding systemic lupus erythematosus (SLE) has evolved dramatically since its first reference during the 1100s, through the neoclassic period of the 1800s when researchers began to refine the definition, and into the modern era with transformative laboratory investigations and discoveries altering the diagnosis and treatment. Weaved into this historic overview are descriptions of the most important contributions of the leading practitioners and researchers.

HISTORIC OVERVIEW: SYSTEMIC LUPUS ERYTHEMATOSUS

Systemic lupus erythematosus (SLE) has a rich historic background. In Latin, the term *lupus* means wolf. During the 1100s, Rogerius Frugardi at the medical school in Salerno, Italy, one of the first medical schools in Western Europe, initially used the term to describe a type of ulcerated skin disease that resembled a 'wolf-eaten' facial rash. For the next 800 years, lupus was used interchangeably to describe any severe deforming dermatologic disorder. See Table 1.

NEOCLASSIC PERIOD

The neoclassical period of lupus history began in the early 1800s and ran into the mid-1900s. During this period order was brought to the field of dermatology.

Robert Willan (1757 to 1812), a dermatologist in Great Britain, is credited with making great strides in classifying skin diseases based on clinical observations. He was the first to clearly differentiate lupus from other disorders.

This classification was further refined by Laurent-Theodore Biett (1781 to 1840), a French physician in Paris, France, who introduced an anatomical approach for analyzing skin disorders. He was the first to accurately describe the cutaneous rash of lupus. But his associate Pierre Louis Alphee-Cazenave (1795 to 1877) is credited with first defining the disease as *lupus erythematosus* in the medical literature.

Ferdinand von Hebra (1816 to 1880), a dermatologist, ran a very prestigious scabies station in Vienna, Austria. He accurately described the discoid and diffuse variance

Disclosures

About this presentation
This presentation was developed from an audio transcript of Prof. Calabrese's presentation at the "Treatment of SLE: Bridging the Gap from Clinical Trials to Practice" symposium held during the Annual Congress of the American College of Rheumatology on November 11, 2012.

The transcript was formatted and edited by Cleveland Clinic and BioMed Central staff for clarity and conciseness, and was then reviewed, revised, and approved by Dr. Calabrese.

Sponsorship
The Cleveland Clinic Foundation Center for Continuing Education acknowledges an educational grant for support of this activity from Human Genome Sciences.

Accreditation
The Cleveland Clinic Foundation Center for Continuing Education is accredited by the Accreditation Council for Continuing Medical Education to provide continuing medical education for physicians. The Cleveland Clinic Foundation Center for Continuing Education designates these (4) enduring activities for a maximum of **1.0** *AMA PRA Category 1 Credit*™. Physicians should claim only the credit commensurate with the extent of their participation in the activity. Participants claiming CME credit from this activity may submit the credit hours to the American Osteopathic Association for Category 2 credit.

To claim CME credit, visit www.ccfcme.org/SLECMEsupplement. CME credit may be obtained upon reading all four CME-certified presentations online.

Author disclosures
In accordance with the Standards for Commercial Support issued by the Accreditation Council for Continuing Medical Education (ACCME), The Cleveland Clinic Foundation Center for Continuing Education requires resolution of all faculty conflicts of interest to ensure CME activities are free of commercial bias. Dr. Calabrese has indicated that he may have relationships which, in the context of his presentation, could be perceived as a potential conflict of interest. Dr. Calabrese has received fees from Amgen, Centocor, Genentech/Roche, and Pfizer for consulting and teaching and speaking; BMS and Savient for consulting; and Sanofi Aventis for consulting and data safety monitoring board.

All other planners, CME staff, and content reviewers, have no relevant financial relationships to disclose.

This presentation has not been subject to peer review. The statements and opinions expressed herein are those of the author, who bears full responsibility for the content of this presentation.

Table 1. Lupus definition timeline

Period	Key investigator	Key contribution
Classic: 1100 to 1800		
c.1100	Rogerius Frugardi	Coined the term *lupus* to describe many types of severe deforming dermatologic disease
Neoclassic: 1800 to mid-1900s		
1700s	Robert Willan	Differentiated lupus from other disorders
c.1800	Laurent-Theodore Biett	Described cutaneous rash of lupus
1800s	Pierre Alphee-Cazenave	Defined lupus erythematosus in literature
1800s	Ferdinand von Hebra	Identified diffuse variants of cutaneous lupus
1800s	Morris Kaposi	Advanced analytic descriptions of lupus
c.1900	William Osler	Introduced concept of systemic, connective tissue disease
1930s to 1940s	Paul Klemperer	Clarified relationship between lupus and several autoimmune disorders
1930s to 1940s	Emanuel Libman	Defined lupus endocarditis
1935 to 1940	George Baehr	Described renal lesions in lupus patients
1930s to 1940s	Arthur Schifrin (with Klemperer)	Major contribution to differential diagnosis of SLE
Modern		
1948	Malcom Hargraves	Identified lupus erythematosus cells in bone marrow samples
1949	John Haserick	Introduced autoimmune concept of lupus

of cutaneous lupus and first used the term *butterfly rash*. His son-in-law Morris Kaposi (1837 to 1902), of Kaposi sarcoma fame, deserves significant credit for his analytical description of patients who probably had lupus erythematosus. He is credited with describing lymphadenopathy arthritis, fever, and many of the systemic manifestations.

Later in this period, between 1888 and 1903, William Osler (1849 to 1919) produced a series of groundbreaking papers describing 28 cases of 'visceral manifestations of erythema group of skin disorders'. He never used lupus to describe these systemic disorders, but he described patients with variant forms of exanthem erythematous rashes and inflammatory purpura. In all likelihood, at least a few of these patients had SLE.

One of Osler's major contributions to this field was his observation that 'this systemic disease that has arthritis, and fever, and lymphadenopathy and other manifestations may occur even in the absence of a rash'. This was a tectonic shift in thinking about connective tissue disease rather than just skin manifestations.

The late neoclassical period is known for the many contributions to pathology and target organ involvement from New Yorkers Emanuel Libman, George Baehr, Arthur Schifrin, and Paul Klemperer. Director of Pathology at Mount Sinai Hospital from 1926 to 1955, Klemperer is credited with clarifying the relationship between cavernomatous transformation of the portal vein and Banti's disease and describing changes of malignant nephrosclerosis. In 1935, Klemperer and Schifrin published a paper on diffuse disease of the peripheral circulation, which made major contributions to the differential diagnosis of SLE. Baehr was the first to describe renal lesions in patients with subacute bacterial endocarditis. Libman was the first to define lupus endocarditis.

Table 2. Therapy timeline

Historical therapies: ~1894 to 1958 to present	
	Antimalarials
	Salicylates
Neomodern therapies: 1952 to present	
	Glucocorticoids
	Alkylator/antimetabolites
Contemporary targeted therapies: 2000 to present	
	B cells
	T cells
	Cytokine based
	Others

MODERN ERA

The modern era began in the laboratory with the discovery of lupus erythematosus cells by Malcom Hargraves. As a pathologist at the Mayo Clinic, Hargraves spent many hours looking at bone marrow cells, and from time to time he would see these extraordinary cells. He differentiated them from tart cells, which were merely phagocytosed nuclei. Careful analyses of many bone marrow samples led to his description, in 1948, of these cells being 'indicative of the disease lupus erythematosus'.

Using bone marrow samples for the diagnosis was a drawback, leading researchers to consider other alternatives. John Haserick, a dermatologist who came to the Cleveland Clinic in 1949, built on this diagnostic work. His discovery that the lupus erythematosus factor induced the lupus erythematosus cell led to the

This presentation has not been subject to peer review. The statements and opinions expressed herein are those of the author, who bears full responsibility for the content of this presentation.

autoimmune concept of lupus. He went on to characterize the lupus erythematosus factor as an immunoglobulin responsible for lupus erythematosus cell phenomena.

Today, SLE is known to range from the organ limited to the diffuse, from the insidious to the fulminate, and from a readily apparent multisystem disease to cases that can only be identified through laboratory tests. These variations collectively contribute to defining disease severity and, ultimately, to defining its response to therapeutic agents. See Table 2.

This presentation has not been subject to peer review. The statements and opinions expressed herein are those of the author, who bears full responsibility for the content of this presentation.
